# Genetic Characterisation of South African and Mozambican Bovine Rotaviruses Reveals a Typical Bovine-like Artiodactyl Constellation Derived through Multiple Reassortment Events

**DOI:** 10.3390/pathogens10101308

**Published:** 2021-10-12

**Authors:** Amy Strydom, Celeste M. Donato, Martin M. Nyaga, Simone S. Boene, Ina Peenze, Milton T. Mogotsi, Eva D. João, Benilde Munlela, A. Christiaan Potgieter, Mapaseka L. Seheri, Nilsa de Deus, Hester G. O’Neill

**Affiliations:** 1 Department of Microbiology and Biochemistry, University of the Free State, Bloemfontein 9300, South Africa; aimster.strydom@gmail.com (A.S.); tmogotsi16@gmail.com (M.T.M.); 2 Enteric Diseases Group, Murdoch Children’s Research Institute, Parkville 3010, Australia; celeste.donato@gmail.com; 3 Department of Paediatrics, Theniversity of Melbourne, Parkville 3010, Australia; 4 Department of Microbiology, Biomedicine Discovery Institute, Monash University, Melbourne 3052, Australia; 5 Next Generation Sequencing Unit, University of the Free State, Bloemfontein 9300, South Africa; nyagamm@ufs.ac.za; 6 Division of Virology, Faculty of Health Sciences, School of Pathology, University of the Free State, Bloemfontein 9300, South Africa; 7 Instituto Nacional de Saúde (INS), Distrito de Marracuene 1120, Mozambique; simonboene@gmail.com (S.S.B.); evadora1@hotmail.com (E.D.J.); benildeantnio@gmail.com (B.M.); ndeus1@yahoo.com (N.d.D.); 8 Biotechnology Center, Eduardo Mondlane University, Maputo 1100, Mozambique; 9 Diarrhoeal Pathogens Research Unit, Department of Virology, Sefako Makgatho Health Sciences University, Pretoria 0001, South Africa; inapeenze@gmail.com (I.P.); mapaseka.seheri@smu.ac.za (M.L.S.); 10 Biochemistry, Focus Area Human Metabolomics, North-West University, Potchefstroom 2520, South Africa; christiaan@Deltamune.co.za; 11 Deltamune (Pty) Ltd., Unit 34 Oxford Office Park, 3 Bauhinia Street, Highveld Techno Park, Centurion 0157, South Africa; 12 Departamento de Ciências Biológicas, Universidade Eduardo Mondlane, Maputo 1100, Mozambique

**Keywords:** bovine rotavirus, artiodactyl genome constellations, A13 genotype, interspecies transmission, transboundary transmission, South Africa and Mozambique

## Abstract

This study presents whole genomes of seven bovine rotavirus strains from South Africa and Mozambique. Double-stranded RNA, extracted from stool samples without prior adaptation to cell culture, was used to synthesise cDNA using a self-annealing anchor primer ligated to dsRNA and random hexamers. The cDNA was subsequently sequenced using an Illumina MiSeq platform without prior genome amplification. All strains exhibited bovine-like artiodactyl genome constellations (G10/G6-P[11]/P[5]-I2-R2-C2-M2-A3/A11/A13-N2-T6-E2-H3). Phylogenetic analysis revealed relatively homogenous strains, which were mostly related to other South African animal strains or to each other. It appears that these study strains represent a specific bovine rotavirus population endemic to Southern Africa that was derived through multiple reassortment events. While one Mozambican strain, MPT307, was similar to the South African strains, the second strain, MPT93, was divergent from the other study strains, exhibiting evidence of interspecies transmission of the VP1 and NSP2 genes. The data presented in this study not only contribute to the knowledge of circulating African bovine rotavirus strains, but also emphasise the need for expanded surveillance of animal rotaviruses in African countries in order to improve our understanding of rotavirus strain diversity.

## 1. Introduction

Rotavirus is an enteric pathogen that affects the young of many mammalian and avian species [1]. The virus belongs to the *Reoviridae* family and contains an 11-segmented double-stranded RNA (dsRNA) genome. The genome encodes six structural proteins that form the viral triple-layered particle (TLP), which consists of the outer capsid (VP4 and VP7), the inner capsid (VP6) and the core (VP2 encasing VP1 and VP3) proteins that enclose the nucleic acid material. The viral genome also encodes five or six non-structural proteins, NSP1-6 [2].

Rotaviruses are classified into nine groups (RVA—RVD; RVF—RVJ) (International Committee on Taxonomy of Viruses (ICTV). Available online: https://talk.ictvonline.org/taxonomy/ (accessed on 20 September 2021)) based on the nucleotide sequence of the VP6 gene. RVA have been extensively studied and a classification system based on the whole genome is used to characterise these viruses. The classification is defined as genotypes Gx-P[x]-Ix-Rx-Cx-Mx-Ax-Nx-Tx-Ex-Hx, where x indicates a numerical number and represents the encoded proteins: VP7-VP4-VP6-VP1-VP2-VP3-NSP1-NSP2-NSP3-NSP4-NSP5/6 [3]. To date, 41 G types and 57 P types have been described in humans and various animal species along with 31 I, 27 R, 23 C, 23 M, 38 A, 26 N, 27 T, 31 E and 27 H types (Rotavirus Classification Working Group: (RCWG). Available online: https://rega.kuleuven.be/cev/viralmetagenomics/virus-classification/rcwg (accessed on 20 September 2021)). Three genogroup constellations, DS-1-like, Wa-like and to a lesser extent AU-1-like are circulating in humans [4]. Analysing the whole genomes of both animal and human RVA indicated that the DS-1-like genotype constellation (G2-P[4]-I2-R2-C2-M2-A2-N2-T2-E2-H2) has a close evolutionary relationship with bovine RVA strains. A typical bovine constellation often presents with G6, G8 or G10 types in combination with P[1], P[5] or P[11] and bovine, DS-1-like and AU-1-like genotypes: I2-R2-C2-M2-A3/A11-N2-T6-E2-H3, as do strains from other species within the mammalian order *Artiodactyla* [4,5].

Rotavirus, which is transmitted via the faecal–oral route, is a common cause of diarrhoea in calves, which can cause economic loss either by mortality or affecting the growth of animals. Severity of disease ranges from asymptomatic carrier animals to mild, self-limiting diarrhoea and, in severe cases, dehydration and death. Mortality is influenced by various factors, including virulence of strains, age of the host and environmental stresses. Although infections are mostly mild, rotavirus is associated with high morbidity [6]. However, in many countries, rotavirus infections in cattle or other animals are not reported. A global review of bovine rotavirus in 24 countries reported prevalence as 33.7% (*n* = 14,076) [7]. The only African countries represented in this study were Tunisia and Nigeria. The majority of bovine rotaviruses had a G6 (50.7%), followed by a G10 (20.6%) G type and the most common P types were P[5] (25.9%) and P[11] (21.5%) [7].

The north-eastern region of South Africa shares a border with the south-western region of Mozambique. Due to the lack of bovine surveillance data, rotavirus prevalence in these countries is not known but a vaccine for cattle, Rotavec^®^ Corona (MSD Animal Health), is available in South Africa. Only limited data for whole genomes of RVA strains originating from various animal species have been described from South Africa. These include strains detected in African buffalo [8], sable antelope [9], horses [10] and cattle species [11,12]. No bovine rotavirus strains have been reported from Mozambique, although a partial strain of possible animal origin was detected in a child, suggesting interspecies transmission [13].

South Africa and Mozambique are both sub-Saharan African countries where rotavirus infections have a major public health impact [14]. Interspecies transmission and reassortment of rotaviruses increase the diversity of the human rotavirus population which can influence vaccine effectiveness. Transboundary transmission of strains between animals will also impact the diversity of rotavirus circulating in a specific population. Characterisation of animal strains is therefore important to aid in the understanding of the genetic diversity of rotavirus strains and their host range specificity. In this study, the whole genomes of bovine rotavirus strains detected in South Africa and Mozambique were characterised.

## 2. Results

### 2.1. Genome Assembly and Genotyping

Genomic data were generated for all seven samples and average coverage for these sequences ranged from 158.2 to 10,555.7 per sequence (Supplementary Materials Table S1). Full-length consensus sequences were assembled for all strains, Bov7, Bov4, MRC-DPRU457, Bov1, MPT93 and MPT307, except for 1162. All the gene sequences of strain 1162 covered at least 80% of the coding region except for the NSP5-encoding sequence, for which only 61.5% of the coding region was obtained (Supplementary Materials Table S1).

The sequences were submitted to GenBank and accession numbers MW771107–MW771172 and MW771184–MW771194 were assigned. The seven bovine strains from Mozambique and South Africa all exhibited artiodactyl bovine-like constellations containing G10/G6-P[11]/P[5]-I2-R2-C2-M2-A3/A11/A13-N2-T6-E2-H3 genotypes (Table 1). Four strains contained G10P[11] genotypes, one strain G6P[11] and the remaining two strains G6P[5]. Variation was also observed for the A genotype (NSP1).

### 2.2. Phylogenetic Analysis

#### 2.2.1. G6 and G10

**Figure 1:**
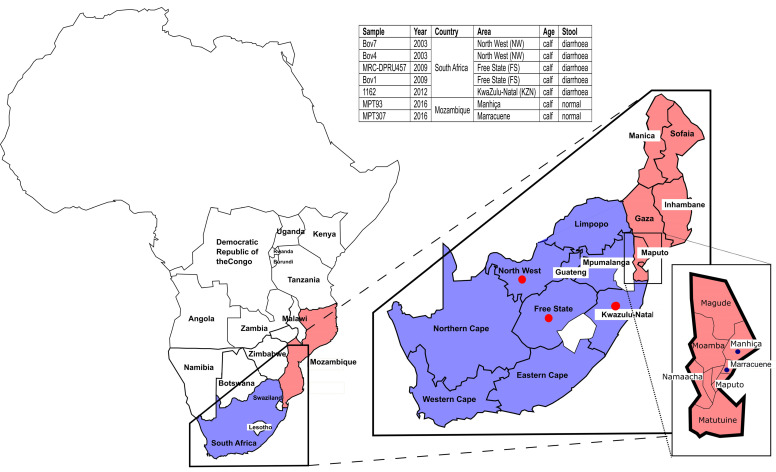
Sampling sites. The map shows South Africa in blue and Mozambique in pink. Sampling sites in South Africa are marked with red circles and in Mozambique with black circles.

**Figure 2:**
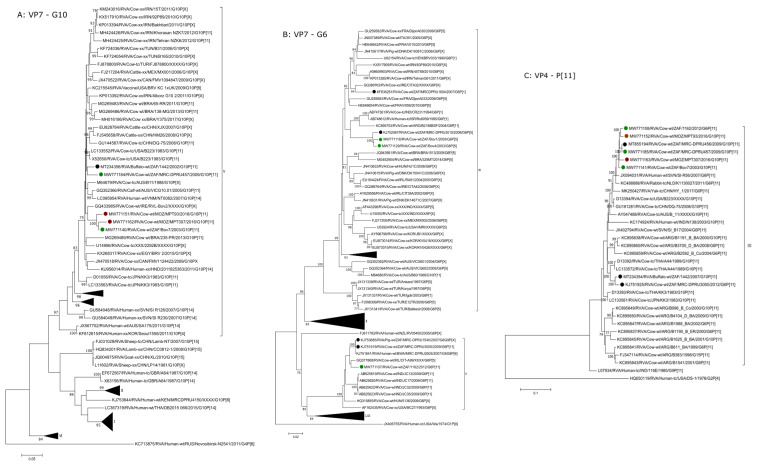

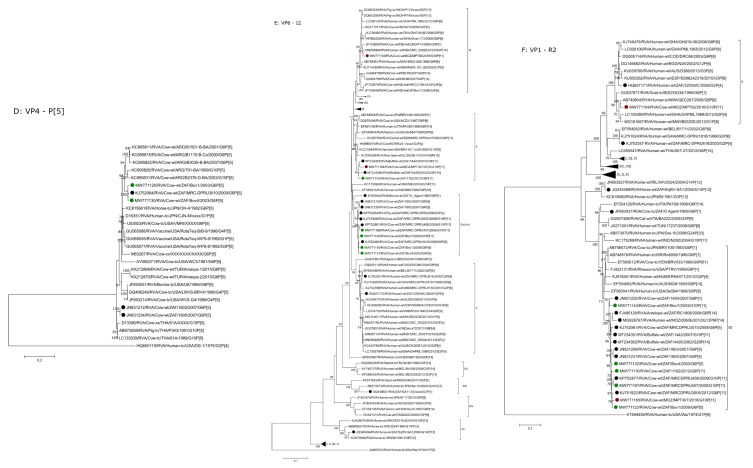

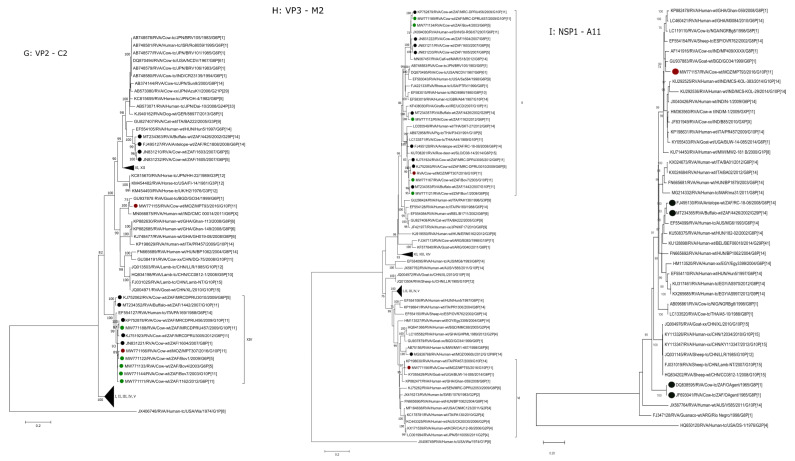

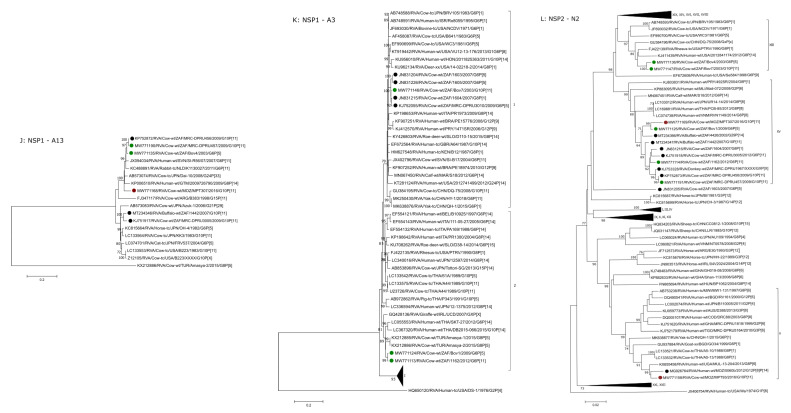

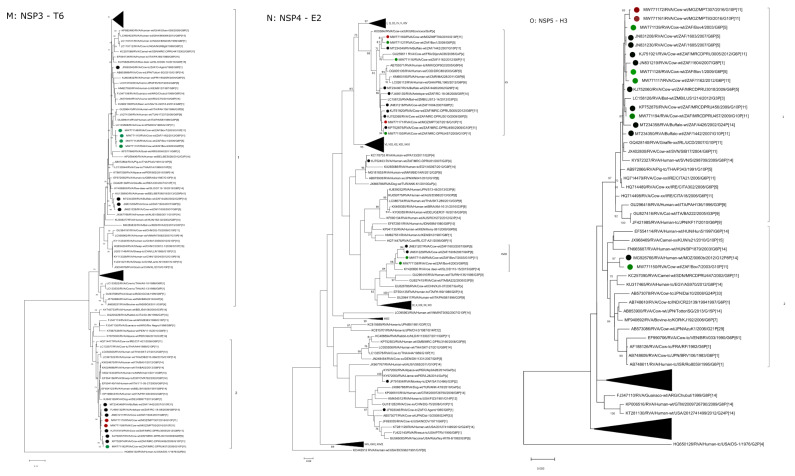
Phylograms based on nucleotide sequences identified for study strains. (**A**): VP7 (G10), (**B**): VP7 (G6), (**C**): VP4 (P[11]); (**D**): VP4 (P[5]), (**E**): VP6 (I2), (**F**): VP1 (R2), (**G**): VP2 (M2), (**H**): VP3 (C2), (**I**): NSP1 (A11), (**J**): NSP1 (A13), (**K**): NSP1 (A13), (**L**): NSP2 (N2), (**M**): NSP3 (T6), (**N**): NSP4 (E2) and (**O**): NSP5 (H3). The South African study strains are indicated with a green circle and the Mozambican study strains are indicated with maroon circles. Previously identified South African and Mozambican strains are indicated with black circles. Each gene was compared with sequences available in GenBank and nucleotide alignments were constructed using the MUSCLE algorithm in the MEGA X [15]. Phylogenetic trees were generated using MEGA X implementing the Maximum Likelihood method and the trees were statistically supported using 1000 pseudoreplicate runs. The optimal model was determined for each gene using JModel Test. For VP7 (G10), VP6 (I2) and NSP4 (E2) the Tamura 3 model + GG4 and for VP7 (G6), VP4 (P[5]), NSP1 (A3) and NSP5 (H3) Tamura 3 + GG4 + I was used. The Tamura-Nei model + GG4 + I was used for VP4 (P[11]) and VP1 (R2). For VP2 (C2), NSP2 (N2) and NSP3 (T6) the General Time Reversible + GG4 and for VP3 (M2) and NSP1 (A11 and A13) General Time Reversible + G_G4_ + I was used. Bootstrap values < 70 are not shown. The trees are drawn to scale, with branch lengths in the same units as those of the evolutionary distances used to infer the phylogenetic tree.

The three G6 bovine strains clustered within lineages IV and V. In lineage IV, Bov1 (detected in the FS in 2009) and Bov4 (detected in the NW in 2003) formed a discrete cluster with the previously characterised, contemporary South African bovine strain, RVA/Cow-wt/ZAF/MRC-DPRU3010/2009/G6P[5] which was detected in KZN in 2009 (Figure 1 and Figure 2). These strains shared 98.68–99.16% nucleotide identity, while the proteins of the respective strains were identical (Supplementary Materials Table S2). The VP7 gene of 1162 (detected in KZN in 2012) shared the highest nucleotide identity (98.44%; 98.92% amino acid identity) with an Irish bovine strain (RVA/Cow-xx/IRE/CITA99/XXXX/G6P[5]) and clustered in lineage V, forming a monophyletic group with MRC-DPRU3005, MRC-DPRU1540 and MRC-DPRU2605.

The four G10 sequences identified in this study formed two different sub-clusters within lineage V (Figure 2). Strain MRC-DPRU457, detected in the FS in 2009, was most closely related to the historic strain RVA/Cow-tc/USA/B223/1983/G10P[11] (96.73% nucleotide and 99.05% amino acid identity) (Figure 1, Supplementary Materials Table S2). The two Mozambican strains detected in 2016 (MPT93 and MPT307) and the South African strain Bov7 detected in 2003 shared 98.00–98.10% nucleotide identity (99.37% amino acid identity) and formed a discrete cluster in the tree. These strains were distinct to most lineage V strains and shared moderate nucleotide identity of 94.19–95.61% (98.21–98.93% amino acid identity) to the most closely related strain, RVA/Cow-wt/IRE/RVL-Bov2/XXXX/G10P[X] (Figure 2, Supplementary Materials Table S2).

#### 2.2.2. P[11] and P[5]

The five P[11] study strains (1162, MPT93, MRC-DPRU457, Bov7 and MPT307) formed a discrete monophyletic cluster in lineage III and shared 96.93–98.46% nucleotide identity (97.98–98.85% amino acid identity). These strains were moderately distinct to global P[11] strains, most closely related to RVA/Human-wt/SVN/SI-R56/2007/G6P[11] and RVA/Rabbit-tc/NLD/K1130027/2011/G6P[11] sharing 96.35–97.84% nucleotide identity (97.98–98.85% amino acid identity). Similar to the G6 VP7 analysis, the two P[5] sequences of Bov1 and Bov4 clustered together with the same South African bovine strain, RVA/Cow-wt/ZAF/MRC-DPRU3010/2009/G6P[5]. These sequences shared 98.46–99.06% nucleotide identity (98.97–99.36% amino acid identity) and were distinct to global P[5] sequences (Supplementary Materials Table S2). Again, the previously characterised strains RVA/Cow-wt/ZAF/1603/2007/G6P[5] and RVA/Cow-wt/ZAF/1605/2007/G6P[5] clustered distinctly from the study strains.

#### 2.2.3. I2

In the VP6 phylogenetic tree, the study strains grouped in three different clusters independent of their G and P genotypes. Four study strains (Bov1, Bov4, Bov7 and MRC-DPRU457) formed a distinct monophyletic cluster with other South African bovine strains, two detected in the Western Cape (RVA/Cow-wt/ZAF/1603/2007/G6P[5] and RVA/Cow-wt/ZAF/1605/2007/G6P[5]) and RVA/Cow-wt/ZAF/MRC-DPRU3010/2009/G6P[5], as well as a South African porcine strain, RVA/Pig-wt/ZAF/MRC-DPRU3878/2008/G5P[X]. MRC-DPRU457 was identical to an unpublished South African strain, RVA/Cow-wt/ZAF/MRC-DPRU456/2009/G6P[11] (Supplementary Materials Table S2). These strains shared 98.49–99.16% nucleotide identity (99.2–99.7% amino acid identity) (Figure 1 and Figure 2, Supplementary Materials Table S2). The VP6 sequences of MPT307 and 1162 clustered with two buffalo strains from South Africa to form a minor South African cluster in lineage X (Figure 2, Supplementary Materials Table S2). These buffalo strains were detected in 2002 and 2007 in Limpopo province [8] (Figure 1). Finally, the VP6 sequence of the Mozambican strain, MPT93, clustered in lineage VI with bovine strains as well as some human strains and shared a 98.74% nucleotide identity (99.5% amino acid identity) with a human strain from India (RVA/Human-wt/IND/CMC_00022/2012/GXP[14]) (Figure 2; Supplementary Materials Table S2). The phylogenetic relationships of this Indian strain in not known. Additionally, the VP6 origins of the human strains in this lineage was inconclusive in previous studies [16,17]. However, the clustering with bovine strains in this study strongly suggests a bovine origin for these human strains.

#### 2.2.4. R2, M2, C2

All the study strains except MPT93 formed a monophyletic group within recognised lineages for VP1 (XII) and VP2 (XIV). The strains were dispersed throughout lineage X in the VP3 tree, clustering with other South African animal strains, as well as a bovine-like Mozambican strain (RVA/Human-wt/MOZ/0060b/2012/G12P[8]P[14]) previously detected in a diarrhetic infant [13]. MPT93 was divergent to other study strains, clustering with a human strain from Malawi, RVA/Human-tc/MWI/QEC287/2006/G8P[8] based on VP1, a goat strain from Bangladesh (RVA/Goat-xx/BGD/GO34/1999/G6P[1]) based on VP2 and a bovine-like human strain from Italy (RVA/Human-wt/ITA/PR457/2009/G10P[14]) based on VP3 (Figure 2).

#### 2.2.5. A13, A11 and A3

Three of the study strains (Bov4, MRC-DPRU457 and MPT307) contained the rarely identified A13 NSP1 genotype. Bov4 clustered with MRC-DPRU457 and RVA/Cow-wt/ZAF/MRC-DPRU456/2009/G6P[11], sharing 98.30% nucleotide identity (98.57% amino acid identity) (Figure 2, Supplementary Materials Table S2). As was seen in the P[11] tree, RVA/Human-wt/SVN/SI-R56/2007/G6P[11] and RVA/Rabbit-tc/NLD/K1130027/2011/G6P[11] clustered with these strains. The Mozambican strain, MPT307 also grouped in the same clade, but shared only 93.80–94.03% nucleotide identity (94.09–94.7% amino acid identity) with the rest of the study strains and 94.30% nucleotide identity with the closest relative, RVA/Cow-tc/JPN/Dai-10/2008/G24P[33]. The Mozambican strain, MPT93, was the only study strain to exhibit an A11 NSP1 gene and shared only 94.35% nucleotide identity (96.74% amino acid identity) with the closest relative (RVA/Goat-wt/BGD/GO34/1999/G6P[1]) (Figure 2, Supplementary Materials Table S2). MPT93 clustered separately from the previously characterised South African buffalo and antelope strains that also exhibit A11 genes. Three study strains (Bov7, Bov1 and 1162) exhibited A3 NSP1 genes (Figure 2). Bov1 and 1162 clustered in a separate clade with bovine-like human G6 and G10 strains from Thailand and bovine G8 strains from Turkey. Bov7 formed a monophyletic cluster with previously characterised South African bovine strains (1605, 1603, MRC-DPRU3010 and 1604) (Figure 2).

#### 2.2.6. N2

The majority of NSP2 sequences of the study strains (Bov1, 1162, MPT307 and MRC-DPRU457) formed a diverse monophyletic group together with previously characterised South African animal strains in lineage XV. Bov7 and Bov4 clustered distinctly in lineage XIII with other diverse bovine strains. MPT93 clustered in lineage V with two bovine-like strains previously detected in humans from Mozambique, RVA/Human-wt/MOZ/0060b/2012/G12P[8]P[14] and Uganda (RVA/Human-wt/UGA/MUL-13-204/2013/G8P[6]), respectively (Figure 1 and Figure 2).

#### 2.2.7. T6

The T6 NSP3 genes of the study strains formed two sub-clusters. MRC-DPRU457 clustered with the two Mozambican strains, MPT307 and MPT93, and formed a diverse monophyletic cluster with previously identified South African animal strains that was distinct from global strains. The study strains shared 97.04–98.68% nucleotide identity (98.68–99.34% amino acid identity) (Supplementary Materials Table S2), whereas Bov1, Bov4, Bov7 and 1162 formed a monophyletic cluster, sharing 95.86–96.93% nucleotide identity (98.49–99.67% amino acid identity) to the closet relative, RVA/Cow-tc/JPN/KK3/1983/G10P[11] (Figure 1 and Figure 2, Supplementary Materials Table S2).

#### 2.2.8. E2

The E2 NSP4 genes of the study strains formed three sub-clusters; two of which fell within lineage XV. MRC-DPRU457 and MPT307 clustered with previously characterised South African animal strains and a bat strain form Zambia in the southern African region. MPT93, Bov1 and 1162 clustered with a South African buffalo strain (RVA/Buffalo-wt/ZAF/1442/2007/G10P[11]) and a French bovine strain (RVA/Cow-xx/FRA/DijonA036/2006/GXP[X]) (Figure 2). These sub-clusters fell within a lineage predominantly comprised of strains from the African continent. Bov7 and Bov4 clustered in lineage XVIII and were closely related to two South African bovine strains previously detected in the Western Cape in 2007 [11] (Figure 1).

#### 2.2.9. H3

All study strains contained a NSP5-encoding gene with a H3 genotype. All strains, except Bov7, clustered together alongside previously characterised South African bovine strains and a Zambian bat strain and shared 98.10–98.92% nucleotide identity (98.36–99.18% amino acid identity) (Supplementary Materials Table S2). Bov7, shared less than 95.40% nucleotide identity (98.4% amino acid identity) with the other study strains and clustered separately, close to a bovine-like strain from Mozambique (RVA/Human-wt/MOZ/0060b/2012/G12P[8]P[14]).

## 3. Discussion

The presence of bovine-like rotavirus strains in humans from Africa has frequently been reported [16,18,19]. However, only a few studies have reported whole-genome characterisation of African bovine rotavirus strains. The whole genomes of three bovine rotavirus strains (RVA/Cow-wt/ZAF/1603/2007/G6P[5], RVA/Cow-wt/ZAF/1604/2007/G8P[1] and RVA/Cow-wt/ZAF/1605/2007/G6P[5]), detected in the Western Cape of South Africa, were described in 2012 [11] and RVA/Cow-wt/ZAF/MRC-DPRU3010/2009/G6P[5], detected in Kwazulu-Natal was reported in 2015 [12]. Two bovine strains, with genotypes G8/G6-P[1]/P[5]-I2-R2-C2-M2-A3/A11-N2-T6-E2-H3, from Nigeria were described in 2016 [20]. Unpublished sequence data for two additional strains, RVA/Cow-wt/ZAF/MRC-DPRU456/2009/G6P[11] and RVA/Cow-wt/ZAF/MRC-DPRU3005/2009/G6P[11], are available in GenBank. The present study adds five more South African bovine strains to this short list, as well as the first two bovine strains detected in Mozambique.

These study strains contained genotypes typically associated with rotavirus detected in bovine hosts: G10/G6-P[11]/P[5]-I2-R2-C2-M2-A3/A11/A13-N2-T6-E2-H3. It is noteworthy that three of the study strains contained the rarely identified A13 genotype adding to the 14 complete sequences in the NCBI GenBank. The only other published study describing an African strain with this genotype was detected in an African buffalo [8]. The study strains had the same genotypes for all segments, except those encoding for VP7, VP4 and NSP1. However, even though the same genotypes were observed for eight of the genes, none of the study strains contained similar sequences across their genomes, indicating that they have probably evolved through various reassortment events and extensive genetic drift. This degree of diversity observed suggests the circulation of multiple, distinct subtypes/alleles in endemic bovine strains in the region that have evolved over decades.

The majority of the study strains almost always clustered with each other or other African animal strains. Of note, study strain RVA/Cow-wt/ZAF/MRCDPRU457/2009/G10P[11] clustered with the contemporary, previously characterised strain RVA/Cow-wt/ZAF/MRC-DPRU456/2009/G6P[11] across all genes analysed, except for the VP7 encoding gene. Bovine (RVA/Cow-wt/ZAF/1603/2007/G6P[5], RVA/Cow-wt/ZAF/1604/2007/G8P[1], RVA/Cow-wt/ZAF/1605/2007/G6P[5], RVA/Cow-wt/ZAF/MRC-DPRU456/2009/G6P[11], RVA/Cow-wt/ZAF/MRC-DPRU3005/2009/G6P[11] and RVA/Cow-wt/ZAF/MRC-DPRU3010/2009/G6P[5]), buffalo (RVA/Buffalo-wt/ZAF/4426/2002/G10P[11] and RVA/Buffalo-wt/ZAF/1442/2007/G10P[11]) and porcine (RVA/Pig-wt/ZAF/MRC-DPRU3878/2008/G5P[X]) strains from South Africa were the most frequent closest relatives to the study strains. These were detected in the Western Cape, Limpopo and North West provinces of South Africa between 2002 and 2009 as well as in Mozambique in 2012. This indicates prolonged and extensive circulation of rotavirus strains throughout the two countries. However, the lack of whole genome data of animal rotavirus strains from South Africa and Mozambique must be noted. Unsampled local diversity is also represented in the long branch lengths in some of the trees and the low nucleotide identity between the study strains and their closest relatives.

In some cases, the study strains clustered with rotaviruses detected in humans from African countries such as the NSP5 sequence of Bov7 which grouped with RVA/Human-wt/MOZ/0060b/2012/G12P[8]P[14] in a separate clade. The majority of the strains in this clade was, however, detected in animals and 0060b was also described as a bovine rotavirus strain detected in a child [13]. This indicates that Bov7 has a bovine-like NSP5 sequence. Similarly, the VP3 sequence of MPT93 clustered in lineage VI with human strains, mostly which are suspected to be a result of interspecies transmission events between humans and cows [21].

The VP1 sequence of MPT93 may also be derived from an interspecies reassortment event as it clustered in lineage V with mostly human strains. The closest relative was a human rotavirus strain, RVA/Human-tc/MWI/QEC287/2006/G8P[8] with a shared nucleotide identity of 98.48% (Figure 2; Supplementary Materials Table S2). This human rotavirus strain was not associated with any interspecies transmission events [16] and the rest of the strains in lineage V were also detected in humans, suggesting that the VP1 sequence of MPT93 is human-like. The NSP2 sequence of MPT93 clustered with strains detected in humans and animals (yak, goat and cow) in lineage V. There is evidence that the NSP2 sequences of the bovine strains (RVA/Cow-tc/THA/A5-10/1988/G8P[1] and RVA/Cow-tc/THA/A5-13/1988/G8P[1]) as well as the goat strain (RVA/Goat-xx/BGD/G034/1999/G6P[1]) are more closely related to human NSP2 sequences [21,22]. This points to a human NSP2 sequence in MPT93; however, the long branch lengths in this lineage suggests unsampled global diversity and increased sequencing of diverse strains from varied hosts may refine the origins of strains in this lineage. Segments VP6, VP2, VP3 and NSP1 of MPT93 clustered with strains from India, Bangladesh and Italy, although the nucleotide identity varied between 94.35 and 98.64%. This indicates that MPT93 is divergent from the rest of the study strains, which are, for the most part, endemic to Southern Africa.

The other Mozambican strain, MPT307, clustered closely to the South African strains for all the segments except that of NSP1-A13, indicating a close relationship between these strains. Additionally, these study strains group closely to strains from other African countries indicating possible transboundary movement of animals and subsequent transmission events. The frequency and extent of transboundary movement in the region is, however, unknown. This observation calls for combined regional efforts between veterinary services to manage infections across international borders.

The limited data available due to local subsampling complicate full characterisation of these study strains but at the same time highlight the significant contribution this study makes to current knowledge about bovine rotaviruses in Africa. Apart from the limited knowledge, the data presented in this study represent a diverse genetic pool of bovine rotavirus strains in Africa that are shaped by extensive reassortment events. These strains circulated for more than a decade in various geographical regions, across country borders. Extensive reassortment is seen, resulting in endemic variants, as well as interspecies transmission in the VP1 and NSP2 genes. Results suggest that transboundary movement and interaction of hosts influence the diversity of rotavirus in Africa. This study calls for extensive surveillance of bovine rotavirus in African countries to understand bovine rotavirus diversity and the extent of interspecies transmission.

## 4. Materials and Methods

### 4.1. Ethical Statement

Collection and testing of bovine faecal samples in South Africa were performed with ethical approval by the Medunsa Research Ethics Committee (MREC/P/103/2008:PG). In Mozambique, the study protocol was approved by the Institutional Bioethics Committee for Health from Instituto Nacional de Saúde (CIBS-INS), reference number 378.050/CIBS-INS/2020.

### 4.2. Samples

Seven rotavirus-positive bovine faecal samples, five from South Africa [(RVA/Cow-wt/ZAF/Bov7/2003/G10P[11] (Bov7), RVA/Cow-wt/ZAF/Bov4/2003/G6P[5] (Bov4), RVA/Cow-wt/ZAF/MRC-DPRU457/2009/G10P[11] (MRC-DPRU457), RVA/Cow-wt/ZAF/Bov1/2009/G6P[5] (Bov1), RVA/Cow-wt/ZAF/1162/2012/G6P[11] (1162)] and two from Mozambique [(RVA/Cow-wt/MOZ/MPT307/2016/G10P[11] (MPT307) and (RVA/Cow-wt/MOZ/MPT93/2016/G10P[11] (MPT93)] were characterised in this study (Figure 1). The South African samples, all diarrhoetic, were submitted to the Diarrhoeal Pathogens Research Unit, Sefako Makgatho Health Sciences University for diagnostic purposes between 2003 and 2012 from the North-West (NW), KwaZulu-Natal (KZN) and Free State (FS) provinces (Figure 1) and tested positive for the presence of rotavirus using the ProSpecT ™ Rotavirus EIA kit (Oxoid, Ely, UK) and electropherotyping. The two Mozambican samples were collected as part of an exploratory study in 2016 from the Manhiça and Marracuene districts in the Maputo province (Figure 1). As it is known that animals can be asymptomatic, samples were collected irrespective of clinical signs (both diarrhoetic and non-diarrhoetic) and sex from newborn to one-year-old animals. Samples were tested for the presence of rotavirus with the ProSpecT ™ Rotavirus EIA kit (Oxoid, Ely, UK) at the Direcção de Ciências Animais (DCA), Directorate of Animal science. The South African samples were taken from commercial herds where all the adult cows had been vaccinated against rotavirus. The Mozambican samples were taken from animals at informal non-commercial smallholdings without any vaccination.

### 4.3. RNA Extraction, cDNA Synthesis and Sequencing

RNA extraction was performed as previously described [23]. Briefly, total RNA was extracted with Tri-Reagent (Sigma) and single-stranded RNA was precipitated with lithium chloride. A self-annealing anchor primer (PC3-T7 loop; Integrated DNA Technologies) was ligated to the dsRNA in order to obtain full-length sequences, with the exception of RVA/Cow-wt/ZAF/1162/2012/G6P[11]. Complementary DNA was synthesised using the Maxima H Minus Double Stranded kit (ThermoFisher Scientific, Waltham, MA, USA). The manufacturer’s instructions were followed with the following modifications. Firstly, the dsRNA was denatured (95 °C; 5 min) immediately before annealing random hexamers and subsequently, first strand synthesis was carried out for two hours at 50 °C.

Sequencing was performed at the University of the Free State Next Generation Sequencing (UFS-NGS) Unit using the Nextera XT DNA Library Preparation Kit (Illumina, Inc., San Diego, CA, USA.) and the MiSeq Reagent Kit V3 (600 cycles).

### 4.4. Maximum Likelihood Phylogenetic Analysis

Sequencing data were assembled as previously described and consensus sequences were analysed using BLASTn [23]. Genotypes were identified using the Rotavirus A Genotype Determination tool available in Virus Pathogen Database and Analysis Resource (ViPR) [24]. Each gene was compared with sequences available in GenBank and nucleotide alignments were constructed using the MUSCLE algorithm in MEGA X [25]. Phylogenetic trees were generated using MEGA X implementing the Maximum Likelihood method and the robustness of branches was assessed by bootstrap analysis using 1000 pseudo replicate runs [25]. The optimal nucleotide substitution model was determined based upon the Akaike information criterion (corrected) (AICc) ranking implemented in jModelTest [26]. For VP7 (G10), VP6 (I2) and NSP4 (E2) the Tamura 3 model + G_G4_ and for VP7 (G6), VP4 (P[5]), NSP1 (A3) and NSP5 (H3) Tamura 3 + GG4 + I was used. The Tamura-Nei model + GG4 + I was used for VP4 (P[11]) and VP1 (R2). For VP2 (C2), NSP2 (N2) and NSP3 (T6) the General Time Reversible + G_G4_ and for VP3 (M2) and NSP1 (A11 and A13) General Time Reversible + G_G4_ + I was used. Lineages for the DS-1-like genotypes [27] and G- and P-genotypes [28] were assigned as previously determined. Nucleotide and amino acid distance matrixes were calculated using the p-distance algorithm in MEGA X.5.

## Figures and Tables

**Table 1:** Artiodactyl bovine-like genome constellations of African bovine rotavirus strains. Top panel: Assigned genotypes. Bottom panel: Assigned lineages (Roman numerals) and clades (brackets).

Strain	VP7	VP4	VP6	VP1	VP2	VP3	NSP1	NSP2	NSP3	NSP4	NSP5/6
RVA/Cow-wt/MOZ/MPT93/2016/G10P[11]	G10	P[11]	I2	R2	C2	M2	A11	N2	T6	E2	H3
RVA/Cow-wt/ZAF/MRC-DPRU457/2009/G10P[11]	G10	P[11]	I2	R2	C2	M2	A13	N2	T6	E2	H3
RVA/Cow-wt/MOZ/MPT307/2016/G10P[11]	G10	P[11]	I2	R2	C2	M2	A13	N2	T6	E2	H3
RVA/Cow-wt/ZAF/Bov7/2003/G10P[11]	G10	P[11]	I2	R2	C2	M2	A3	N2	T6	E2	H3
RVA/Cow-wt/ZAF/1162/2012/G6P[11]	G6	P[11]	I2	R2	C2	M2	A3	N2	T6	E2	H3
RVA/Cow-wt/ZAF/Bov4/2003/G6P[5]	G6	P[5]	I2	R2	C2	M2	A13	N2	T6	E2	H3
RVA/Cow-wt/ZAF/Bov1/2009/G6P[5]	G6	P[5]	I2	R2	C2	M2	A3	N2	T6	E2	H3
RVA/Cow-wt/MOZ/MPT93/2016/G10P[11]	G10 V	P[11] III	VI	V	Distinct	VI	A11	V	T6 (1)	XV	H3 (1)
RVA/Cow-wt/ZAF/MRC-DPRU457/2009/G10P[11]	G10 V	P[11] III	Distinct?	XII	XIV	X	A13	XV	T6 (1)	XV	H3 (1)
RVA/Cow-wt/MOZ/MPT307/2016/G10P[11]	G10 V	P[11] III	X	XII	XIV	X	A13	XV	T6 (1)	XV	H3 (1)
RVA/Cow-wt/ZAF/Bov7/2003/G10P[11]	G10 V	P[11] III	Distinct?	XII	XIV	X	A3 (1)	XIII	T6 (2)	XVIII	H3 (2)
RVA/Cow-wt/ZAF/1162/2012/G6P[11]	G6 V	P[11] III	X	XII	XIV	X	A3 (2)	XV	T6 (2)	XV	H3 (1)
RVA/Cow-wt/ZAF/Bov4/2003/G6P[5]	G6 IV	P[5]	Distinct?	XII	XIV	X	A13	XIII	T6 (2)	XVIII	H3 (1)
RVA/Cow-wt/ZAF/Bov1/2009/G6P[5]	G6 IV	P[5]	Distinct?	XII	XIV	X	A3 (2)	XV	T6 (2)	XV	H3 (1)

## Data Availability

The nucleotide sequences generated in this study were submitted to GenBank and accession numbers MW771107–MW771172 and MW771184–MW771194 were assigned.

## Supplementary Materials

The following are available online at https://www.mdpi.com/article/10.3390/pathogens10101308/s1, Supplementary Table S1: Assembly; Supplementary Table S2: Distance matrixes.
